# Common Sense Beliefs about the Central Self, Moral Character, and the Brain

**DOI:** 10.3389/fpsyg.2015.02007

**Published:** 2016-01-12

**Authors:** Diego Fernandez-Duque, Barry Schwartz

**Affiliations:** ^1^Psychology Department, Villanova UniversityVillanova, PA, USA; ^2^Psychology Department, Swarthmore CollegeSwarthmore, PA, USA

**Keywords:** lay theories, common-sense beliefs, true self, self-concept, materialism, brain-mind relation, essentialism

## Abstract

To assess lay beliefs about self and brain, we probed people's opinions about the central self, in relation to morality, willful control, and brain relevance. In study 1, 172 participants compared the central self to the peripheral self. The central self, construed at this abstract level, was seen as more brain-based than the peripheral self, less changeable through willful control, and yet more indicative of moral character. In study 2, 210 participants described 18 specific personality traits on 6 dimensions: centrality to self, moral relevance, willful control, brain dependence, temporal stability, and desirability. Consistent with Study 1, centrality to the self, construed at this more concrete level, was positively correlated to brain dependence. Centrality to the self was also correlated to desirability and temporal stability, but not to morality or willful control. We discuss differences and similarities between abstract (Study 1) and concrete (Study 2) levels of construal of the central self, and conclude that in contemporary American society people readily embrace the brain as the underlying substrate of who they truly are.

## Introduction

How much does your brain contribute to your core self? Can you willfully change who you *truly* are? Should your moral character be judged by core attributes of your self which you cannot willfully control? Science might 1 day provide answers to these fascinating questions, but for the purpose of our study, those scientific answers are beside the point. Instead, we set to uncover the *common sense* beliefs that people hold at the intersection of these three domains: “brain,” “self,” and “morality.” We explore the perceived relation of the brain to the constructs of “central self,” “moral character,” and “willful control,” to better understand the impact of neuroscience discourse on current lay theories of the mind. More to the point, we explore the possibility that in current American society people acknowledge that the brain is the underlying substrate of who they *truly* are, as well as the implications of this for lay theories of morality and free will.

### Lay theory of the self

In all aspects of cognition, from perception to decision making, people are confronted with an amount and complexity of information that far exceeds their computational abilities. This is particularly true of the social world. People handle this problem by relying on common sense beliefs about how the world works. Those beliefs are structured into semantic networks known as lay theories, which help ordinary folk in making inferences and explaining phenomena^1^.

Starting with the seminal work of Heider in 1958, psychologists have described several domains of knowledge in which people organize information with the help of “lay theories.” These include a theory of physics (McCloskey et al., 1983), a theory of biology (Slaughter et al., 1999), a theory of mind (Astington and Baird, 2005), and a theory of mental disease (Haslam, 2005; Ahn et al., 2006), as well as a theory on the essence of natural kinds (Gelman, 2004), and another one on the malleability of human intelligence (Dweck, 2008). There is a lay theory of morality and free will (Nahmias et al., 2005; Monroe and Malle, 2010), and also a lay theory of the mind/brain relation (Bloom, 2004); and of course, there is a lay theory of the self, which we describe in the next paragraphs.

First of all, people think of themselves not as a disparate collection of thoughts and dispositions, but rather as a cohesive unit somewhat stable over time, especially as it projects into the future (Neisser, 1988; Moore et al., 2001; Quoidbach et al., 2013). In other words, people see themselves as a “self”^2^. Furthermore, people make a distinction between the core self (sometimes referred to as a person's “true self” or “essence”) and the more peripheral aspects of the self (sometimes referred to as the “superficial” self) (Johnson et al., 2004). People think of the central self as being at the core of who they are; the central self defines them (Schlegel et al., 2011). As such, the lay person thinks of the central self as mostly virtuous (Newman et al., 2014) and stable over time (Wakslak et al., 2008). Scientists draw the same distinction in their own construct of the self, which they see as hierarchically organized, with some traits being central and others being more peripheral (Markus and Wurf, 1987; Sedikides, 1995).

The self is sometimes construed at an abstract level (who I am), and at the more concrete level of personality traits (kind, intelligent). At the concrete level, personality traits are sometimes thought of as the building blocks of the self; some traits are seen as more stable, essential to who a person is, and thus at the core of personal identity (Haslam et al., 2004). In particular, moral traits such as honesty and kindness are deemed essential to personal identity, in the sense that people believe that losing those traits makes you a different individual (Strohminger and Nichols, 2014). Other traits are deemed more peripheral (Markus and Wurf, 1987; Sedikides, 1995). Relative to peripheral traits, central traits are deemed more relevant for impression formation, that is, they are the traits one would most like to know when judging an unfamiliar person (Asch, 1946). Not surprisingly, when asked to reflect on their own identity, people judge their central traits more favorably than their peripheral traits (Alicke and Sedikides, 2009; Newman et al., 2014). If asked to name the attributes that best describe the central self, items like *honesty* and *kindness* often come to mind (Goodwin et al., 2014). Traits like these reveal moral character and perhaps for that reason people sometimes think of them as willfully controlled (Goodwin et al., 2014, but see Johnson et al., 2004).

In sum, central traits can be defined as personally relevant attributes at the core of who the person believes herself to be, so that without those attributes she would be a different person. In contrast, peripheral traits are traits that people see as descriptive of them but not so much defining who they are.

### Lay theory of mind/brain relation

At least since the time of Descartes (1984/1641), people have thought of themselves as bodies and minds. As bodies, people were part of the natural world and bound by the laws of nature. But as minds, they were exalted—capable of reason, moral evaluation, appreciation of beauty and of sacredness, and free will. Today, most people still believe both in moral responsibility and free will (Nahmias et al., 2005; Monroe and Malle, 2010) holding others responsible for actions only in situations where a choice to act differently was available (Greene and Cohen, 2004; Mele, 2009; Monroe and Malle, 2010). But the explosion of research in neuroscience, with descriptions in the popular media of brains doing things that minds were supposed to do, may have begun to challenge the picture of minds and brains that we inherited from Descartes.

Over the last few decades, researchers have begun to explore what ordinary folk think about the relation between brain and mind. Some of the evidence points toward common sense dualism, the folk view that the mind and the brain are separate entities (Bloom, 2004). Many religious beliefs that are popular across the world, such as beliefs in the afterlife and in the existence of the soul, suggest a dualist concept of mind/brain relation (Bering and Bjorklund, 2004). And developmental research suggests that children start as dualists, and become materialists only years later—if at all—through formal education (Johnson and Wellman, 1982; Hood et al., 2012; Forstmann and Burgmer, 2015). However, other research suggests that in Western cultures the mind is often identified with the brain (Johnson, 1990; Lillard, 1998). When asked “Do you need the brain to ____?” both adults and elementary school children endorse the view that the brain is necessary for all sorts of human psychological activities, from perception to emotions to cognitive acts such as *reading* and *writing*. In other words, when asked about the *functions* of mind and brain, elementary school children and adults alike treat the brain as responsible for the functions of the mind. One way to reconcile these contradictory findings is to argue that people admit the brain's contribution to these psychological processes, but reserve a special non-material place for who they *truly* are (Bloom, 2004).

In other words, folk theory may admit neural contributions to narrowly defined psychological processes such as seeing, thinking, and problem-solving, but reject that who a person *truly* is (i.e., the core self) depends on the brain.

### Lay theory of morality in its relation to the brain

Researchers have also begun to assess folk beliefs about the relations among brain, moral responsibility, and free will (Greene and Cohen, 2004). In one study, the presence of brain images nudges participants to endorse deterministic explanations of criminal behavior and minimize moral condemnation, even when the images are not explicitly related to the case with textual elaboration (Beall et al., 2013). In another example, expert testimony on the neurobiological mechanisms of psychopathy causes judges to consider those mechanisms as mitigating factors, thus leading to reduced criminal sentences (Aspinwall et al., 2012). Reading research about the neural bases of human behavior leads people away from retributive punishment presumably because learning about neuroscience highlights a mechanistic worldview in which free will is diminished, and therefore actors should be held less blameworthy for their acts (Monterosso et al., 2005; Shariff et al., 2014). And yet, the influence of neuroscientific determinism on moral evaluation is far from absolute. For example, some research suggests that determinism undermines free will in the abstract, but does not excuse wrongdoing in concrete cases; people might think “The world may be fully deterministic in which case you are not responsible for your acts, but if you steal my wallet, that is still inexcusable” (Nichols, 2011). Other research suggests that people embrace both determinism *and* free will, a position known as *compatibilism*. Thus, when morally evaluating an action, people sometimes state that even if the universe is fully deterministic, the actor could act differently (Nahmias et al., 2005; Monroe and Malle, 2010). Other times, when presented with neuroscience claims that “free will does not exist because choices are caused by neural impulses” people reply by appealing to a different level of analysis, focusing on the agent to state that “the person makes the neural impulses happen.” People may endorse the neuroscientific character of psychological states and acts without committing to a deterministic view of them.

In sum, there is much variability in people's judgments about these relations, and internally inconsistent responses abound.

### Probing background beliefs with a less intrusive, non-experimental approach

Most previous research has used an experimental approach, manipulating the saliency of neuroscience, and probing perceived free will and moral responsibility of hypothetical acts. Our research took a different approach; rather than priming certain properties and probing hypothetical acts, we simply asked participants about their self and about their personality traits. In doing so, we hoped to probe stable beliefs rather than beliefs triggered by the specific context imposed by the experiment. Given the inconsistency, if not incoherence, in people's beliefs about agency and responsibility as revealed by past research, it seemed to us possible that the answers people give to probes about agency and responsibility in specific contexts may not reveal much about the ideas people carry around with them when they are not challenged by puzzling and problematic cases.

Our approach also allowed us to assess the pattern of relations at different levels of construal for the concept of the self. There is some evidence that at higher levels of construal, people conceptualize the self as more abstract and stable (i.e., closer to the concept of the *true* self). In those cases, people expect their distant-future self to be more consistent with their true self (Wakslak et al., 2008). This raises the possibility that probing the self at a high level of construal would give rise to a different pattern of relations between its properties (i.e., brain dependence, willful control, temporal stability, moral relevance) than asking about those properties at the level of specific personality traits.

### Overview of current studies

In two studies, we assessed people's beliefs about the properties of the self, particularly its relation to the brain, morality, and willful control. In study 1, participants compared the “Central Self” (“the person you *truly* are”) and the “Peripheral Self” (“the set of things that describe you but don't define you”) in their brain dependence, moral relevance, willful control, and temporal stability. In Study 2, participants judged specific personality traits (e.g., kindness, humor, etc.) on their centrality to the self, brain dependence, moral relevance, willful control, and temporal stability. We assessed the following hypothesis.

H1. Participants will reject the brain as the underlying substrate of who they truly are. That is, they should judge the Central Self (the *true* self) as less dependent on the brain than the Peripheral Self (Study 1), and they should judge traits deemed most central to the self as least dependent on the brain (Study 2).

Furthermore, we explored whether probing the self at a high level of construal (Study 1) may give rise to a different pattern of relations between its properties (i.e., brain dependence, willful control, temporal stability, moral relevance), than asking about those properties at the level of specific personality traits (Study 2). In particular, participants may be willing to acknowledge the brain contribution to the central self when conceptualized at the concrete level of personality traits, but not at a more idealized, abstract level.

## Study 1: The central self abstractly construed

### Method

#### Disclosure of research conduct and IRB approval

For this and all the other experiments, we report all measures collected, all data exclusions (if any), and all manipulations. The project was reviewed and approved by the Institutional Review Board (IRB) of Villanova University (project 13-077).

##### Participants

A total of 172 participants were recruited via mturk and paid US$3.50 to complete the survey. We recruited only from people residing in the United States, with a record of diligent performance in mturk surveys, as determined by a “hit” approval rate higher than 97%. Other demographic information appears in Table 1. No participant reported having done a similar task before.

**Table 1:** **Demographic Information for Study 1 and 2**.

	**Study 1**	**Study 2**
% men	43.2	53.3
Age (SD, range)	33.1 (SD = 11; 18-72)	34.1 (SD = 12; 19-68)
Average household size	2.7	2.8
**% OF Ss IN EACH INCOME BRACKET**		
Less than 25 k	18	18
25–50 k	35	35
50–75 k	22	25
>75k	25	22
**% OF Ss BY EDUCATION**		
High school diploma	35	39
Bachelor	55	51
higher degree	11	10
**RELIGIOSITY (1 = NOT RELIGIOUS, 7 = DEEPLY RELIGIOUS)**		
Men	2.8 (SD = 2.0)	2.6 (SD = 2.1)
Women	3.1 (SD = 2.2)	3.4 (SD = 2.2)
**% OF PEOPLE IDENTIFYING WITH EACH AN ORGANIZED RELIGION**		
None	46	49
Protestant/Christian	28	28
Catholic	15	16
Other	12	7

#### Materials and procedure

Participants who signed on via mturk to do the study were confronted with a series of questions implemented using Qualtrics. The first question was an Instructional Manipulation Check, aimed at ensuring that participants were reading the instructions thoroughly (Oppenheimer et al., 2009).

After answering the Instructional Manipulation Check, participants were asked to reflect for a minute about “What kind of person am I? What am I like?” More precisely, they were presented with the following prompt:

**What kind of person am I? What am I like?** Think for a minute about what you are like as a person. Personal attributes could include aspects of your personality, your skills and abilities, your aspirations, your beliefs, your behaviors, your interactions with others, your emotional tendencies.

After that initial prompt, participants were randomly assigned to one of two orders of task presentation. Order A started with a relative comparison of the Central vs. the Peripheral Self; order B started with separate assessments of Central and Peripheral Self. In order A, which was completed by 85 participants (49.4%), the following screen introduced the concepts of Core Self and Peripheral Self:

**The CORE SELF, who you *truly* are**. Some of those personal attributes you thought about are at the core of who you are, so that if you lacked those attributes you would be a different person. That is your core self.

**The PERIPHERAL SELF, things that describe you but don't define you**. Other personal attributes you thought about apply to you but do not so much define you, so that if you didn't have those attributes, you would still be the same person. That is your peripheral self.

After reading these definitions, participants judged the relative contribution of the brain to the core and peripheral self, by answering the following question:

**Which is more brain-based: your core self or your peripheral self?** Some people like to say that “the mind is what the brain does.” However, it is possible that some mental attributes are more brain-based than others. Would you say that the brain is more responsible for the CORE attributes of your self or for the PERIPHERAL attributes of your self?

Participants answered by moving a slide switch horizontally along a 100-point scale that was anchored at −50 with the label “Core Self” and at +50 with the label “Peripheral Self.” The slide switch was always centered at the 0-point mark at the time of its initial presentation; its movements were displayed analogically (i.e., the location of the switch on the scale).

Subsequently, participants made judgments on the moral relevance, willful control, temporal stability, and desirability of the central vs. peripheral self. The questions were displayed sequentially in fixed order. The exact wording was:

In judging your MORAL CHARACTER, what should people rely on more: the CORE aspects of your self or the PERIPHERAL aspects of your self?

Which do you think would be easier for you to change through WILLFUL CONTROL, if so you wanted: the CORE attributes of your self or the PERIPHERAL attributes of your self?

Which personal attributes do you think will still be part of your self three years from now: those currently at the CORE of your self, or those currently at the PERIPHERY of your self?

Which one contains more positive or desirable qualities, your CORE self or your PERIPHERAL self?

Which one contains more negative or undesirable qualities, your CORE self or your PERIPHERAL self?

The response slider remained on display throughout the survey but the arrowhead was reset to the midpoint (“0”) for each question; a reminder of the definition of core and peripheral self was displayed as at the top of each screen throughout the survey.

After completing these relative judgments, participants in order A moved to assess the Core Self separately: They were reminded one more time of the concept of the core self, and asked to make judgments about it. The exact wording was:

**How much does the brain contribute to the core aspects of your self?** This question has led to great debate among researchers. In your opinion: (0 = The brain contributes NOTHING to your core self; 100 = The brain contributes EVERYTHING to your core self).

**What do the core aspects of your self reveal about your moral character?** In other words, how much should people rely on those core attributes when judging your moral character? In your opinion: (0 = your core self is not at all indicative of your moral character; 100 = your core self is completely indicative of your moral character).

**Are the core aspects of your self under your willful control? In other words, could you change those core attributes if you really wanted to?** (0 = NOT AT ALL under control; 100 = COMPLETELY under control).

**Are the core aspects of your self stable over time**, so that three years from now they will still describe you to the same extent as they do now? (0 = Variable throughout the years; 100 = stable throughout the years).

**How positive are the core aspects of your self?** (0 = Mostly Negative; 100 = Mostly Positive).

For these questions, the scale always ranged from 0 to 100, with the slide switch initially centered at the 50 point mark. Finally, participants assessed the Peripheral Self separately, using the same format and procedure. Participants in order B (*n* = 87; 50.6%) started by evaluating the Central Self and the Peripheral Self separately, and then moved to make the relative comparison of the Central vs. the Peripheral Self.

The next step for all participants was to list 5 traits central to their sense of self and 5 peripheral ones; we would later compare these answers to the traits we had selected for Study 2. The general instructions for this section were as follows:

“What kind of person am I? What am I like?” Name 10 personal attributes that describe what you are like. Include items that are central to who you are, but also include items that are somewhat peripheral to your sense of self (5 central, 5 peripheral). Remember, a central attribute is one that is at the core of who you are so that if you lacked this attribute, you would be a different person. A peripheral attribute is one that applies to you but doesn't so much define who you are so that if you didn't have this attribute, you would still be the same person. You are welcome to list any item that you honestly believe properly describes what you are like (there is no fixed menu of items to select from). If you are not sure where to start, you may want to think about things such as: aspects of your personality, your skills and abilities, your aspirations, your beliefs, your behaviors, your interactions with others, your emotional tendencies, etc.

After those general instructions, participants were asked to fill in a table entitled “Attributes describing the kind of person that I am.” The table had two columns (central, peripheral) and 5 rows. A message at the top of the screen reminded participants “In the spaces below, type 10 personal attributes that describe what are you like. Include items that are central to who you are, but also include items that are somewhat peripheral to your sense of self (5 central, 5 peripheral).”

Next, participants provided demographic information (age, gender, household income in US dollars, household size, level of education, and religiosity). Finally, to explore possible correlations between the perceived contribution of the brain to the self, and people's general beliefs about mind/brain relation and free will, we asked participants to complete two questionnaires: a 27-item Dualism Scale (Stanovich, 1989) and the Free Will and Determinism Plus (FAD-P) scale (Paulhus and Carey, 2011). The FAD-P was completed by a subset of participants (129 out of a total of 172; 75%). The dualism scale developed by Stanovich (1989) probes many versions of dualism and materialism. There are items on Cartesian dualism (“the mind and the brain are two totally separate things”), popular dualism (“minds are inside brains but are not the same as brains”), and property dualism (“knowledge of the mind will forever be beyond the understanding of the sciences like physics, neurophysiology and psychology”), as well as statements highlighting the existence of introspection (“the ‘self’ I introspect about controls the mind and the brain”). The items on materialism include statements on identity theory (“for each thought that I have there exists a certain state that my brain is in”), and on eliminative materialism (“just as we no longer talk about witches, in the future when we know in detail how brains work we may not talk about minds anymore”). The FAD-P contains four relatively independent subscales. The Free Will subscale probes lay beliefs about people's capacity for free action (e.g., “People have complete control over the decisions they make.” “Strength of mind can always overcome the body's desires”). The other three subscales probe Scientific Determinism (e.g., “Your genes determine your future”), Fatalistic Determinism (e.g., “My future has already been determined by fate”), and Unpredictability (e.g., “Life is hard to predict because it is almost totally random”).

### Results

Ninety-two percent of participants (159/172) answered the Instructional Manipulation Check successfully, suggesting they were reading the instructions thoroughly. The 13 participants who failed the instructional manipulation check were warned about their mistake and encouraged to provide a new answer after reading the instructions more carefully. They all answered correctly the second time. Every single participant answered correctly all other six instances of the Reading Manipulation Check that were interspersed throughout the study. Thus, we did not exclude any participants from the analysis.

Table 2 shows the mean responses on the relative comparison of Central Self vs. Peripheral Self. Not surprisingly, the Central Self was judged to be more positive than the Peripheral Self, and also more indicative of moral character, as revealed by one sample *t*-tests against zero (i.e., center of the scale). Importantly, the Central Self was judged to be more brain-based than the Peripheral Self. The Central Self was deemed harder to change through willful control, and more stable over the years.

**Table 2:** **Mean scores on relative comparison of central self vs. peripheral self in Study 1**.

	**Central vs. peripheral self**		
	**Mean (SD)**	**95% CI**	***t***
Which one is more brain-based?	−15.4 (28.7)	−19.7, −11.1	−7.0
In judging your character, which one should we rely on?	−26.3 (26.3)	−29.9, −22.7	−14.3
Which one is easier to change through willful control?	25.9 (25.0)	22.2, 29.7	13.6
Which attributes will be part of you 3 years from now?	−30.5 (25.8)	−34.4, −26.6	−15.5
Which one contains more desirable qualities?	−18.0 (25.6)	−21.2, −14.1	−9.2
Which one contains more undesirable qualities?	11.6 (23.4)	8.0, 15.1	6.4

The scale was anchored in −50 (Central Self) and +50 (Peripheral Self); one sample t-tests against zero, df = 171, all tests significant at p < 0.001.

Table 3 shows the mean responses in the assessment of the Central Self and the Peripheral Self when asked separately. For each question, we ran a paired-samples *t*-test that compared the judgment on the Central Self to the judgment on the Peripheral Self. The pattern of results in this dataset matched perfectly the pattern of results for relative comparisons depicted in Table 2. Not surprisingly, the Central Self was judged more positively than the Peripheral Self, and more indicative of moral character. The brain contribution was deemed larger for the Central Self than for the Peripheral Self, and large for both of them (i.e., above the 50% mark). The Central Self was once again judged to be harder to change through willful control, and the Central Self was deemed more stable over the years than the Peripheral Self.

**Table 3:** **Mean scores on the separate assessment of central self and peripheral self in Study 1**.

	**Central self Mean (SD)**	**Peripheral self Mean (SD)**	**95% CI**	***t***	***F***
Brain contribution	76.5 (20.8)	66.0 (22.6)	5.9, 15.2	4.5	12.0
Moral relevance	81.0 (17.8)	50.0 (24.1)	26.6, 35.4	13.8	137.7
Willful control	56.7 (27.1)	71.9 (22.6)	−20.4, −9.9	−5.7	22.3
Temporal stability	70.9 (23.1)	39.5 (25.2)	26.6, 36.1	13.1	128.9
Desirability	76.5 (17.7)	67.0 (17.6)	6.5, 12.5	6.2	32.7

Paired-samples t-tests, df = 171, t-values; ANCOVAs controlling for “psychology” covariate, df = 165; all tests significant at p < 0.001.

It is possible that the core self was judged more brain based simply because the peripheral self contains more non-psychological attributes (e.g., being tall). We controlled for this possible artifact in the data analysis, by including a “psychology” covariate. For this, we first looked at the attributes that participants reported as relevant to their self, and coded each attribute as being either “psychological,” “ambiguous,” or “not psychological.” A total of 168 out of 172 participants provided complete data. The items were coded by a research assistant blind to the purpose of the study, and by the study's first author (Cronbach's alpha = 0.81). “Psychological” was defined as “things you need a mind for”: illustrative examples for the blind coder were “kind” and “perseverant,” as opposed to “androgynous” and “beach bum” which were examples of “ambiguous,” and “tall” which was a non-psychological example. Among the 53 most frequently mentioned items (i.e., those mentioned by at least 5 participants) the vast majority were “psychological” (50/53). The exceptions were “overweight” and “athletic” which were judged as “not psychological,” and “hardworking,” which was judged as “ambiguous” by one of the coders. Ninety-two percent of responses to items mentioned as central to the self (774/840) and 78.5% of responses to items listed as peripheral attributes (659/840) were deemed to be psychological by the study's first author (86 and 69% respectively by the second coder). We used these data to compute the number of psychological attributes each participant listed as central to the self, minus those listed as peripheral. We included that “psychology” gap as a covariate and ran for each question a one-way repeated measures ANCOVA with two levels (Central, Peripheral). All the effects were highly significant (ps < 0.001) even after controlling for this possible artifact (see F values at Table 3, right column).

Finally, we assessed whether either the perceived brain contribution to the Central Self (“how much does the brain contribute to the core aspects of your self?”) and/or the perceived brain contribution to the peripheral self (“how much does the brain contribute to the peripheral aspects of your self?”) were correlated with any of the individual differences measures, including the dualism scale, the four subscales of the Free Will and Determinism scale (FAD-P), religiosity, and education. We found that the belief that the central self was brain based was negatively related to dualism, *r*_(160)_ = −0.23, *p* = 0.003, and positively related to the Scientific Determinism subscale of the Free Will and Determinism Scale, *r*_(126)_ = 0.21, *p* = 0.02. There were no other significant correlations involving perceived brain contribution (see Table 4).

**Table 4:** **Pearson product-moment correlations between the brain contribution, individual differences measures, and demographics (Study 1)**.

	**1**	**2**	**3**	**4**	**5**	**6**	**7**	**8**	**9**	**10**
1. Brain, central self	–	−0.01	−0.23^**^	0.04	0.21^*^	−0.13	0.00	−0.12	0.04	0.03
2. Brain, peripheral self		–	−0.13	−0.08	−0.13	0.07	0.00	0.02	−0.03	−0.03
3. Dualism scale			–	0.28^**^	−0.31^**^	0.25^**^	0.02	0.43^**^	−0.07	−0.02
4. FAD, free will				–	−0.13	−0.12	0.17	0.25^**^	0.08	0.01
5. FAD, scientific determinism					–	0.15	0.22^*^	−0.16	0.09	−0.04
6. FAD, fatalistic determinism						–	0.01	0.41^**^	−0.04	−0.08
7. FAD, unpredictability							–	−0.09	0.05	0.20^*^
8. Religiosity								–	0.09	−0.05
9. Education									–	0.30^**^
10. Income										–

* p < 0.05,

** p < 0.01, two-tailed.

### Discussion

Participants in Study 1 readily accepted the brain's contribution to the self. This was the case for the peripheral self, and even more so for the central self. In a direct comparison, the brain was deemed more responsible for the core attributes of the self than for the peripheral ones. As expected, the central self was deemed more indicative of moral character and more stable across time. Interestingly, the central self was also seen as less changeable through willful control.

## Study 2: The self construed at the level of personality traits

In Study 2, we assessed the same relations as in Study 1, but with the self construed at the more concrete level of personality traits. Personality traits constitute the building blocks of people's concept of who they are—their self-concept. In Study 2, we selected 18 personality traits that people often list as important to their self-identity and asked participants to evaluate each of them on six dimensions. Central traits were defined as personally relevant attributes at the core of who the person believes herself to be, so that without those attributes she would be a different person. In contrast, peripheral traits were defined as traits that people see as descriptive of them but not so much defining who they are. We had participants assess the centrality of the trait to the self, how brain based it was, how revealing it was of moral character, how changeable it was by willful control, how stable it was, and how desirable it was.

### Method

#### Pilot study

In a pilot study, 29 participants recruited via mturk were asked first to list five personal strengths and five weaknesses, and later to sort them into central and peripheral attributes to the self. They offered attributes like “intelligent,” “caring,” “honest,” “funny,” and “shy.” Another 29 participants were asked first to list five central traits and five peripheral traits, and although they disproportionately named strengths instead of weaknesses, and their responses were somewhat more varied, the content of the two lists overlapped.

#### Main study

##### Participants

A total of 210 new participants were recruited via mturk and paid US$3.50 to complete the survey, using the same criteria as in Study 1. All but one person reported not having done a similar task before (when asking this question, we reassured participants that their payment would not be jeopardized by their answer). Table 1 shows demographic information. All but 3 participants provided demographic information; their profiles looked similar to those of participants in Study 1, with the exception that in Study 2 there were more men than women.

##### Stimulus development

We used the results of the pilot study to construct the stimulus materials for the main study, which consisted of nine positive and nine negative personality traits (for a complete list, see Table 5). At that time, we had not yet conducted Study 1 and therefore did not have data from that study to help us in the construction of the stimulus materials for Study 2. Nonetheless, we later conducted an item analysis to confirm that the traits used in Study 2 were comparable to the traits listed by participants of Study 1.

**Table 5:** **Mean scores and 95% confidence intervals for each dimension for each trait of Study 2**.

	**Brain**	**Central to Self**	**Moral**	**Control**	**Stable**	**Desirable**
Intelligent	91 [89, 93]	81 [79, 83]	28 [24, 33]	46 [42, 51]	81 [78, 84]	94 [93, 96]
Creative	82 [79, 85]	68 [64, 72]	25 [21, 30]	42 [37, 46]	73 [70, 76]	87 [85, 90]
Organized	72 [69,76]	62 [58, 66]	33 [28, 37]	78 [76, 81]	69 [66, 73]	87 [85, 89]
Optimistic	72 [68, 75]	66 [62, 70]	49 [45, 54]	67 [63, 71]	62 [58, 66]	85 [83, 88]
Funny	68 [65, 72]	67 [64, 70]	29 [25, 33]	56 [52, 60]	70 [67, 73]	85 [83, 87]
Honest	66 [61, 70]	83 [81, 86]	92 [90, 94]	86 [83, 88]	73 [70, 77]	94 [92, 95]
Hardworking	63 [59, 70]	75 [72, 78]	69 [65, 73]	84 [82, 87]	71 [67, 74]	92 [90, 94]
Kind	62 [58, 66]	80 [77, 82]	86 [84, 89]	80 [77, 83]	70 [67, 74]	92 [90, 94]
Loyal	58 [54, 63]	81 [78, 84]	82 [78, 85]	78 [75, 81]	72 [68, 75]	89 87, 92]
Anxious	76 [72, 79]	41 [37, 46]	20 [17, 24]	42 [38, 46]	50 [46, 54]	14 [11, 16]
Pessimistic	69 [65, 73]	33 [29, 37]	42 [37, 47]	63 [59, 67]	54 [50, 58]	14 [12, 17]
Judgmental	65 [61, 70]	36 [32, 40]	67 [63, 71]	71 [67, 75]	57 [53, 60]	19 [16, 22]
Shy	65 [61,69]	51 [47, 56]	20 [17, 24]	46 [42, 50]	59 [55, 63]	22 [19, 25]
Impatient	64 [59, 68]	40 [36, 43]	38 [34, 42]	65 [61, 69]	55 [51, 59]	13 [11, 15]
Disorganized	63 [59, 67]	31 [27, 36]	25 [21, 29]	74 [70, 77]	53 [49, 57]	10 [08, 13]
Aloof	62 [58, 66]	32 [28, 36]	36 [32, 41]	63 [59, 67]	55 [51, 59]	21 [18, 24]
Selfish	60 [56, 65]	28 [24, 32]	75 [71, 78]	77 [73, 80]	52 [48, 56]	11 [08, 13]
Lazy	54 [49, 58]	33 [29, 37]	50 [46, 55]	79 [76, 82]	49 [45, 53]	8 [06, 10]

N = 210; a 100-point scale anchored with “not at all” at 0, and “completely” at 100; Labels: Brain, Brain contribution; Moral, Morally Relevant; Control, Under Willful Control; Stable, Stable Across Time.

Out of 172 participants in Study 1, 168 provided traits. There were 5 central traits and 5 peripheral traits per participant, for a total of 1620 items. Among the 10 traits most often listed as central to the self in Study 1, there were six of the nine positive traits assessed in Study 2 (honest, kind, intelligent, loyal, hardworking, creative). A seventh trait also made the list when aggregated with its synonym (funny, humorous). The other four traits most frequently mentioned as central to the self in Study 1 were close semantic associates to traits presented in Study 2. For example, one frequently mentioned trait was *smart*, which semantic memory studies show is the first word that comes to mind when one hears the word *intelligent*, which was listed in Study 2. Other frequently mentioned traits in Study 1 were *caring, loving*, and *compassionate*, all of which are semantically related to the word *kind*, listed in Study 2 (Nelson et al., 1998). Only two of the nine positive traits of Study 2 were not mentioned frequently by Study 1 participants as central to their self (optimistic, organized). Excluding those items from the analysis leaves the results of Study 2 unchanged. Thus, it seems that, by and large, the positive items listed in Study 2 captured well the traits that participants in Study 1 associated with their Central Self.

In Study 1, negative traits were mentioned more often as part of the Peripheral Self than as part of the Central Self. Thus, to assess whether the negative traits used in Study 2 matched those of Study 1, we started by exploring the Peripheral Self list. Among the 10 negative traits most frequently listed in the Peripheral List in Study 1, there were six of the nine negative traits of Study 2 (lazy, anxious, shy, impatient, selfish, judgmental). These same six items sat at the top of the list of negative traits mentioned for the Central Self. Thus, it seems that six of the negative items of Study 2 were well represented in Study 1. The other three items (aloof, disorganized, pessimist) were mentioned much less often. Excluding those items from the analysis leaves the results of Study 2 unchanged.

##### Procedure

The first question was the Instructional Manipulation Check aimed at ensuring that participants were reading the instructions thoroughly. Next were the general instructions: “You will see a list of 18 personal attributes, displayed one at a time. They include aspects of personality, skills and abilities, interactions with others, emotional tendencies, etc. For each attribute, answer all 6 questions on the page.” Following, participants saw 18 traits randomly displayed and answered six questions, which were presented one at a time in a fixed order. The first question probed the relevance of the trait to the participant's own self-concept as follows:

**How CENTRAL is this attribute to who you are as a person?** Is this an attribute that does not even apply to you at all? Is it a peripheral attribute that applies to you but doesn't so much define who you are, so that if you didn't have this attribute, you would still be the same person? Or is this an attribute at the core of who you are, so that if you lacked this attribute, you would be a different person?

**Figure:**
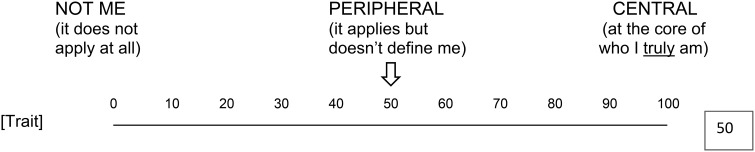


Participants answered by moving a slide switch horizontally along a 100-point scale. The scale was anchored with the label “NOT ME (it does not apply at all)” at 0 and the label “CENTRAL (at the core of who I *truly* am)” at 100. The slide switch was centered at the 50-point, which was labeled “PERIPHERAL (it applies but doesn't define me)”; its movements were displayed analogically (i.e., the location of the switch on the scale) and digitally (i.e., a number informing the exact location of the switch in the 100-point range). Each trait remained on display throughout all six questions, and on every presentation the arrowhead and the digital box were reset to the midpoint (“50”); the response slider remained on display throughout the survey.

The next five questions asked participants to reflect on the trait regardless of whether they themselves possess it. These questions probed the perceived contribution of the brain to the trait and the moral relevance, willful control, temporal stability, and desirability of the trait, as follows:

**How much does the BRAIN contribute to this attribute in those who have it?** This question has led to great debate among researchers. In your opinion, this attribute is: (0 = not at all due to the brain; 100 = completely due to the brain)

**How much does this attribute reveal about the MORAL CHARACTER of a person?** How much should we rely on this attribute when judging a person's character? (0 = not at all indicative of moral character; 100 = completely indicative of moral character)

**How much is this attribute under WILLFUL CONTROL?** Is this something one could change if one really wanted to? (0 = not at all controllable; 100 = completely controllable)

**How STABLE is this attribute in a person's life?** For example, will someone who is described by this attribute today also be described by it to the same extent 3 years from now? (0 = not at all stable; 100 = completely stable)

**How DESIRABLE is this attribute?** How much is this attribute a weakness one might wish weren't true rather than a strength one might feel proud of? (0 = very undesirable; 100 = very desirable)

Next, participants provided demographic information (same as in Study 1). Finally, to explore possible correlations between the perceived contribution of the brain to personality traits, and people's general beliefs about mind/brain relation and free will, we asked participants to complete two questionnaires: all participants completed a 27-item Dualism Scale (Stanovich, 1989), and some participants (*n* = 123) also completed the Free Will and Determinism Plus (FAD-P) scale (Paulhus and Carey, 2011). The whole survey took approximately 25 min.

### Results and discussion

Ninety percent of participants (188/210) answered the Instructional Manipulation Check successfully, suggesting they were reading the instructions thoroughly. The 22 participants who failed the instructional manipulation check were warned about their mistake and encouraged to provide a new answer after reading the instructions more carefully. They all answered correctly the second time. Thus, we did not exclude any participants from the analysis.

Table 5 shows the average response across the 210 participants for each dimension of each of the 18 traits. For every trait, the brain's contribution was deemed larger than 50%, although, as expected, there was a wide range, from 54% for laziness to 91% for intelligence. As intended, all positive traits were deemed desirable (>85 points on a 100-point scale), and all negative traits were deemed undesirable (< 23 points). Interestingly, while many of the traits central to the self were moral traits (e.g., honest, kind, loyal), some of them were not (e.g., intelligent).

To analyze the data, we first standardized the variables. Next, we used R (R Core Team, 2014) and *lme4* (Bates et al., 2015) to perform linear mixed effects modeling, with cross-random effects for participants and traits (Judd et al., 2012). For example, to explore the relation between “centrality to the self” and “perceived brain contribution,” we entered “brain contribution” as the outcome variable and self-centrality as the fixed effect. As random effects, we included the intercepts for subjects and traits, as well as the by-subject and by-trait random slopes for the effect of self-centrality. In other words, we ran the following model:

$$\begin{array}{l} {\text{Brain}}_{\text{ijk}}=\beta_{\text{ojk}}+\beta_{1\text{jk}}*\left({\text{SelfCentrality}}_{\text{ij}}\right)+{\text{r}}_{\text{ijk}} \end{array}$$

Where i = scores, j = participants, k = traits, β_ojk_ = α + η_j_ + η_k_, and β_ijk_ = α + ε_j_ + ε_*k*_

This model accounted for the fact that the data in our study were nested not only within individuals but also nested within traits (i.e., data were cross-classified). Furthermore, the inclusion of the random intercept for traits and the by-trait random slope accounted for the fact that the traits we chose for the study were a sample of a larger universe. To establish statistical significance, *p*-values were obtained by likelihood ratio tests of the full model with self-centrality against the model without self-centrality. The model was fitted using restricted maximum likelihood estimation (REML). Self-centrality affected perceived brain contribution, χ^2^(1) = 15.5, *p* < 0.0001. We repeated this approach for each pair of dimensions. The results are summarized in Table 6, where we report the regression standardized coefficients (β), standard errors (SE), and *p*-values.

**Table 6:** **Linear mixed effect modeling of Study 2 data, with cross-random effects for participants and traits**.

**Outcome variable**	**Predictor variable**	**β**	**SE**	**χ^2^**	***p*-value**
Brain	Central to self	0.10^**^	0.022	15.5	< 0.001
	Desirable	0.07	0.04	3.6	0.06
	Stable	0.11^**^	0.022	25.2	< 0.001
	Control	−0.01	0.03	0.2	0.63
	Moral	−0.03	0.03	1.0	0.3
Moral	Central to self	0.03	0.04	0.6	0.4
	Desirable	0.11	0.11	1.1	0.29
	Stable	0.04	0.02	2.7	0.1
	Control	0.19^**^	0.02	34.1	< 0.001
Control	Central to self	0.01	0.03	0.6	0.3
	Desirable	0.07	0.1	0.4	0.5
	Stable	−0.09	0.03	6.6	0.01
Stable	Central to self	0.16^**^	0.02	29.7	< 0.001
	Desirable	0.33^**^	0.04	28.7	< 0.001
Central to self	Desirable	0.59^**^	0.06	34.0	< 0.001

** p < 0.01.

For the “self-centrality” dimension, a theoretically interesting question lay in the “within-individuals” relation: would traits seen as relatively central to the self by a participant also be seen as relatively desirable by *that* participant? To address this question, we computed within-participant correlations between self-centrality and each of the other dimensions, and averaged those correlations across subjects. These results largely replicated the pattern obtained with the mixed effect modeling (r_desirable_ = 0.64, r_stable_ = 0.37, r_control_ = 0.04, r_moral_ = 0.17, r_brain_ = 0.17).

Finally, we assessed possible correlations between perceived contribution of the brain to personality traits, and people's general beliefs about mind/brain relation and free will in Stanovich's Dualism Scale and Pauhlus and Carey's FDA-P scale. For each participant, we calculated the average score across traits to the question “How much does the brain contribute to this trait?” and correlated this score with each of the questionnaires. The belief that traits were brain based was negatively related with dualism, *r*_(208)_ = −0.21, *p* = 0.002, 95% CI [−0.30, −0.05], and with religiosity, *r*_(208)_ = −0.17, *p* = 0.02, 95% CI [−0.30, −0.04]. There were no significant correlations between the belief that traits were brain based and any of the four subscales of the Free Will and Determinism scale (Paulhus and Carey, 2011). Unexpectedly, the belief that traits were brain based was negatively correlated with level of education *r*_(208)_ = −0.15, *p* = 0.04, 95% CI [−0.28, −0.02].

In sum, in Study 2, centrality to the self was positively correlated to brain contribution. Centrality to the self was also correlated with desirability and temporal stability; interestingly, centrality to the self was not related to moral character nor to willful control. In fact, some of the traits central to the self were non-moral and also unchangeable through willful control (e.g., “intelligent”). Finally, traits deemed changeable through willful control were deemed more informative of moral character than unchangeable traits (see Table 7).

**Table 7:** **Pearson product-moment correlations between the brain contribution, individual differences measures, and demographics (Study 2)**.

	**1**	**2**	**3**	**4**	**5**	**6**	**7**	**8**	**9**
1. Brain, peripheral self		−0.21^**^	0.14	0.04	−0.12	0.13	−0.17^*^	−0.15^*^	0.12
2. Dualism scale			0.22^*^	−0.31^**^	0.42^**^	−0.09	0.46^**^	−0.16^*^	0.00
3. FAD, free will				0.13	0.12	0.22^*^	0.15	−0.26^**^	0.04
4. FAD, scientific determinism					0.16	0.33^**^	−0.14	0.05	−0.05
5. FAD, fatalistic determinism						0.16	−0.48^**^	−0.11	−0.07
6. FAD, unpredictability							−0.09	−0.11	−0.07
7. Religiosity								−0.01	0.02
8. Education									0.12
9. Income									

* p < 0.05,

** p < 0.01, two-tailed.

## General discussion

In two studies, we probed the concept of the self to explore the pattern of relations among its properties (i.e., brain contribution, willful control, temporal stability, moral relevance). In both studies, the brain's contribution was deemed larger for the Central Self than for the Peripheral Self. Thus, our findings show that in current American society, people readily acknowledge the contribution of the brain to the Central Self (against our Hypothesis). Future studies should seek to extend these findings to other cultures where construal of the self may differ, and more thoroughly explore the possible moderating role of religiosity and socioeconomic status (Markus and Kitayama, 1991, 2010).

By and large, the results were consistent across the two studies. Both the Central Self *per se* (Study 1) and traits central to the Self (Study 2) were seen as most desirable and stable across time, and as already mentioned, more dependent on the brain. However, some differences did emerge. For example, only at the abstract level of construal did participants endorse the view that the central self (i.e., the true self) was less changeable through willful control than the peripheral self and yet more revealing of moral character. What could explain this paradox? One likely explanation is that when reasoning about the central self at the abstract level, there are some answers that appear obvious, but in fact are mutually inconsistent:

1. The “central self” is who “you truly are” and as such it is less amenable to change; it is under lesser willful control (essences are fixed and stable).
2. Your central self is who “you truly are” and as such it is more revealing of your moral character than the peripheral self. Consistent with these first two claims, participants believe that “the best indicators of the authentic self are not the products of personal choice but reactions over which the individual may exert relatively little voluntary control” (Johnson et al., 2004, p. 627).
3. Moral character is determined by behavior that is under the willful control of the actor. Although we did not assess this statement in our study, we take it to be non-controversial, as it is a widespread common sense belief that guides much of the psychology of blame (Alicke, 2000; Monterosso et al., 2005).

These three statements are mutually incoherent because if your moral character depends on things you can control (3) and your moral character is informed by your central self (2) then it follows that your central self should be under willful control (against 1). However, taken in isolation, each statement seems uncontroversial. At the abstract level of construal, participants seem to answer these questions independently, disregarding the inconsistency across questions. It is also possible that participants conceptualized the central self as a collection of both moral and non-moral traits. If so, they might have felt entitled to report that the central self was not amenable to change because non-moral traits such as intelligence are considered fixed.

The traits used in Study 2 matched fairly well with the traits that participants in Study 1 reported spontaneously to describe the self. In both studies, the central self was thought of as a collection of both positive moral traits (e.g., honest, kind, loyal) and some non-moral traits (e.g., intelligence). The association between the central self and morally relevant traits has often been reported in the literature (Goodwin et al., 2014); in contrast, the inclusion of non-moral traits as part of the central self is less well established, and future studies should further explore it by probing a larger sample of traits. Studying a larger number of traits would also test the generalizability of the current findings.

More broadly, the results of our study speak to the relation between mind and brain in folk theory. The literature in cognitive development suggests that children espouse commonsense dualism, and that monism is a cultural artifact of formal education (Wellman, 1990; Bloom, 2004). According to this view, people learn in school and through the internet and other media that “the brain underlies the mind” the same way that people learn all sorts of strange, unintuitive scientific facts. Contrary to this view, we failed to find reliable correlations between level of education and dualism, as measured not only by questions about the brain contribution to the self but also by a well-validated dualism scale (Stanovich, 1989). Admittedly, in the current study the correlations among individual difference measures of lay beliefs were modest at best. Thus, conclusive answers on this issue should await further exploration. Besides, our study was limited to a very specific cultural group and it would be interesting to explore how its results compare to other cultures' folk theories of the mind. Finally, an interesting topic that remains to be addressed is the study of the mechanisms and processes by which scientific ideas about mind/brain relation become spread to society at large^3^.

## Conclusions

In sum, we have found that in current American society, people readily acknowledge the contribution of the brain to the Central Self, both when construed abstractly and when construed at the level of personality traits. We think these findings are a step forward in describing what ordinary folk think to be the properties of the self, in the spirit of a “common sense” psychology that can be traced to social psychologists such as Heider (Heider, 1958; Malle, 2008). That said, how people think about self, brain, agency, and responsibility may evolve as people have more contact with research in neuroscience. As the paradox of seeing the central self as most morally relevant and yet least changeable through willful control illustrates, the current lay understanding of self, brain and moral agency is unsettled. Our research can be seen as a still image of a process that is likely to be dynamic. Time will tell how the movie unfolds.

## Author contributions

DF and BS designed research; DF performed research; DF analyzed data; and DF and BS wrote the paper.

## Data accessibility

The data for both studies can be accessed at Open Science Framework, at the link https://osf.io/3pv92/ (Fernandez-Duque, 2016).

### Conflict of interest statement

The authors declare that the research was conducted in the absence of any commercial or financial relationships that could be construed as a potential conflict of interest.

## Footnotes

^1^Some argue that humans are endowed by nature with this core knowledge, citing as evidence its presence at a very early age and its universal development (Wellman and Gelman, 1998). Others favor instead the view that lay theories are social constructions, and point to cultural differences as evidence of it. A prime example of this latter view is Social Representation Theory which claims that “our social representations come from the world of science communicated to us through the mass media and elaborated upon by ordinary people to help make sense of everyday life” (Rateau et al., 2011, p. 478). Despite their differences, both positions share the view that information is organized as a “structure,” with some core elements providing more stable, abstract meaning. For Social Representation Theory in particular, these core elements are more fully shared among members of the culture.

^2^A historically interesting illustration of this point is Descartes' famous inference “Cogito ergo sum” (*I* think, therefore *I* am) which some critics have argued goes beyond what is justified by assuming there is a self (“I”) doing the thinking. Those critics would prefer the statement to be “thinking is occurring.” Whatever philosophical merits that criticism might have, it is Descartes' rendition that best aligns with common sense in the Western World. Buddhism, on the other hand, subscribes to the idea that there is no “self,” but rather a sequence of phenomena passing by (Arnold, 2012).

^3^Much has been written on the popularization of scientific ideas (Moscovici, 1961/2008). As for the specific reasons that explain the spread of this idea in western society, one contributing factor is likely to be the admiration that society affords to neuroscience, relative to the low status it gives to psychology (Keil et al., 2010; Fernandez-Duque et al., 2015). Another contributing factor is likely to be the metaphoric understanding of these abstract concepts, in ways that allow for productive mapping between them (Lakoff and Johnson, 1980; Fernandez-Duque and Johnson, 2002).
